# Prognostic impact of coronary microvascular dysfunction in patients with myocardial infarction evaluated by new angiography-derived index of microvascular resistance

**DOI:** 10.1016/j.ijcha.2024.101575

**Published:** 2024-12-05

**Authors:** Benoit Caullery, Laurent Riou, Stephanie Marliere, Estelle Vautrin, Nicolas Piliero, Olivier Ormerzzano, Helene Bouvaist, Gerald Vanzetto, Gilles Barone-Rochette

**Affiliations:** a Department of Cardiology, University Hospital, 38000 Grenoble, France; b University Grenoble Alpes, INSERM, CHU Grenoble Alpes, LRB, 38000 Grenoble, France; c French Clinical Research Infrastructure Network, 75018 Paris, France

**Keywords:** STEMI, Coronary physiology, Coronary microvascular dysfunction, AngioIMR

## Abstract

**Background:**

Several methods for measuring IMR derived from angiography have been developed. AngioIMR is a novel method for the assessment of angiography-derived IMR with no requirement for a wire and hyperemia. The prognostic value of AngioIMR is unknown in STEMI patients. We aimed to provide the prognostic value of AngioIMR in patients with ST-elevation myocardial infarction (STEMI).

**Methods:**

This study included patients with STEMI who underwent invasive coronary angiography and primary percutaneous coronary intervention (PPCI). AngioIMR was calculated using computational flow and pressure simulation immediately after PPCI. The presence of significant coronary microvascular dysfunction was defined as AngioIMR > 40. The primary outcome was a composite of all cause death or hospitalization for heart failure (MACE).

**Results:**

A total of 178 patients were included (65.0 ± 12.8 years on average, 74 % male gender). An AngioIMR > 40 was found in 72 patients. During a median follow-up of 2.9 (2.3–6.9) years, a primary endpoint was observed in 56 patients. By Kaplan-Meier analysis, the risk of MACE was significantly higher in patients with AngioIMR > 40 (log-rank P < 0.01). An Angio IMR > 40 was significantly associated with the occurrence of the primary endpoint in univariate (70 % vs 27 %; hazard ratio 4.519; 95 % CI: 2.550–8.009; p < 0.0001) and multivariate analysis (Hazard ratio 4.282; 95 % CI: 2.325–7.886; p < 0.0001). AngioIMR model showed incremental prognostic value compared to a model with clinical and imaging risk predictors (C-index 0.84 vs 0.79; p = 0.04).

**Conlusion:**

Elevated AngioIMR showed a independent prognostic significance in STEMI patients. In addition to well-known risk factors, assessment of coronary microvascular dysfunction can be a feasible approach for early prevention and a therapeutic target in STEMI patients.

## Graphical abstract

**Figure:**
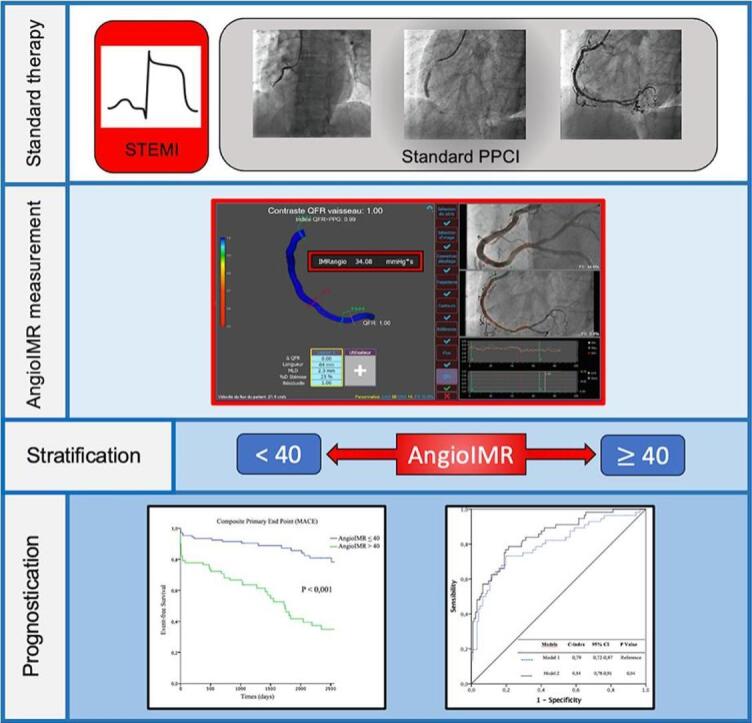

## 1 Introduction

In ST-segment elevation myocardial infarction (STEMI), primary percutaneous coronary revascularization (PPCI) is the standard treatment [1]. Restoration of normal flow indicates the success of the procedure. However, only 50 % of patients have complete revascularization when considering coronary flow at the microcirculatory level [2], [3]. Coronary microcirculatory dysfunction (CMD) has a poor prognosis. Its noninvasive and indirect assessment may be performed using cardiac magnetic resonance imaging (CMR) through the evaluation of microvascular obstruction (MVO) [4], [5], [6]. However, the 24–48 h gap between PPCI and MVO evaluation hinders the timely detection of suboptimal revascularization in the catheterization laboratory, which in turn delays early intervention in cases of less than optimal microvascular revascularization.

Intracoronary physiological evaluation can also be performed during the initial coronary catheterization. Invasive measurement of the index of microcirculatory resistance (IMR) allows reliable quantification of CMD, independently of the epicardial network or hemodynamic state [7], [8], [9]. Several ultrasound, positron emission tomography (PET) or CMR studies have shown the relationship between IMR and left ventricular recovery, or infarct size [10], [11], [12]. Finally, Fearon et al demonstrated the independent prognostic value of an IMR > 40 in relation to all cause death or hospitalization for heart failure [13]. Nevertheless, this invasive technique comes with significant limitations. It requires expertise, specialized equipment, a substantial procedure cost, an extended procedure duration, the induction of hyperemia with specific drug-related complications, and most importantly, the insertion of a pressure wire with rares but serious complication like coronary dissection [14].

Advancements in technology have now made it feasible to acquire physiological measurements derived from angiography without the need for a pressure wire or inducing hyperemia [15]. Several angiogram-based IMR indices have been devised, with some necessitating hyperemia induction and others feasible at rest. A novel non-invasive IMR, referred to as AngioIMR, has been introduced, which can be performed at rest while simulating hyperemia [16]. The diagnostic efficacy of this index has been validated against IMR measurements obtained with a pressure wire in chronic coronary syndrome patient population. However, no prognostic data are available to date in STEMI patients.

Accordingly, the aim of this study is to evaluate the prognostic value of AngioIMR in patients with STEMI.

## 2 Methods

### 2.1 Study population

**Fig. 1:**
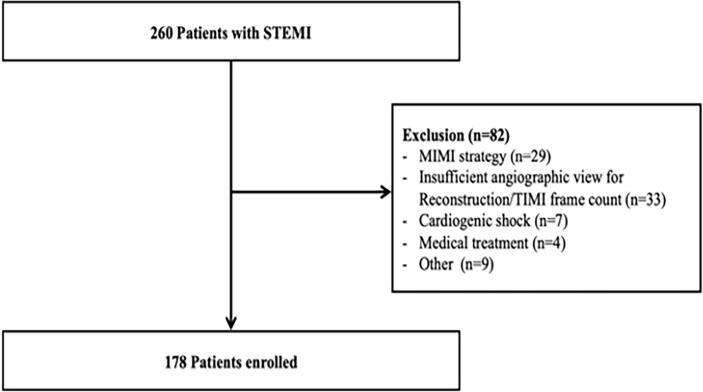
**Flow chart** STEMI = ST elevation myocardial infarction; MIMI = Minimalist immediate mechanical intervention; TIMI = Thrombolysis in myocardial infarction.

The present study is an observational, retrospective cohort study which is set up for the development of software using artificial intelligence. The study population was composed of patients diagnosed with STEMI [17] and selected from a previous independent study cohort who underwent PPCI at the Grenoble university hospital center (FRANCE) between January 2015 and August 2018 (AIDECORO; NCT: 04598997). Patients who required extracorporeal life support, Impella or intra-aortic balloon counter pulsation were not included. The others exclusion criteria were: age < 18 years, refusal to participate in the study, a delayed stenting strategy, post-PPCI thrombolysis in myocardial infarction (TIMI) flow < 2, technical limitations preventing the assessment of AngioIMR (absence of additional incidence, artifact, poor image quality). Thus, 260 patients with STEMI and successful revascularization were selected. After exclusion, 178 patients were selected for the present study (Fig. 1). Each subject signed informed consent; the study was performed considering the Helsinki Declaration and was approved by the Hospital Review Committee.

### 2.2 Invasive coronary angiography

Patients were administered 250 mg of aspirin intravenously and either 600 mg of clopidogrel, 60 mg of prasugrel, or 180 mg of ticagrelor before cardiac catheterization. Administration of glycoprotein (GP) IIb/IIIa inhibitors, balloon predilatation, and manual thrombus aspiration were performed at the treating physician's discretion. Procedural anticoagulation consisted of unfractionated heparin. A drug eluting stent was implanted following appropriate sizing.

All patients were treated with aspirin (75 mg/day) indefinitely and an adenosine diphosphate receptor antagonist for 1 year. High-dose statins, betablockers, and angiotensin-converting enzyme inhibitors or receptor blockers were also prescribed in the absence of contraindications in accordance with European guidelines [1].

### 2.3 Angiography derived IMR (AngioIMR)

Intracoronary nitroglycerin was administered following PPCI and AngioIMR was measured. Routine coronary angiograms were performed using rapid contrast injection over three cardiac cycles and a frame rate of 15 frames per second for AngioIMR measurement. The best end-diastolic frame with good contrast opacification and minimal overlap was selected in both projections. AngioIMR calculation was performed using the QAngio XA 3D software (QFR®, version 2.1 Research Edition, Medis Medical Imaging, Leiden, the Netherlands) as previously described [16] and blinded from the clinical data. Briefly, the following steps were performed: (**a**) selection of 2 angiographic image sequences > 25° apart; (**b**) selection of proper contrast-filled end-diastolic frames; (**c**) identification of anatomical landmarks for automated correction of system distortions; (**d**) definition of start and end points followed by delineation of the lumen in the 2 projections; (**e**) TIMI contrast frame count was carried out manually and the mean resting aortic pressure (Pa) was introduced as input; and finally **(f)** 3D reconstruction of the lumen and the reference surface with the QFR value and angioIMR.

AngioIMR was computed using the following formula:

AngioIMR=(e-Pahyp)∗QFR∗(vessellength/Vhyp)

Pa was the mean aortic pressure in resting conditions; based on previous data a correction factor to estimate Pahyp (e-Pahyp) from resting Pa (Parest) was applied [18].

e-Pahyp=Parest-[0.1∗Parest]

Vessel length was defined by the vessel segment used for QFR evaluation, and the hyperemic coronary flow velocity (Vhyp) was modeled by applying a quadratic function over resting frame count analysis [19]. An Angio-IMR > 40 was used to define CMD. All analyses were blinded to the outcome results. The analysis (B.C) was performed with the vendor’s certified specific software that had been used for training.

### 2.4 Data collection and outcomes

Patients were prospectively followed. A self-administered questionnaire was sent, and non-responders were contacted by telephone interviews. If they remained unresponsive, their cardiologists or general practitioners were reached. The duration of follow-up was calculated starting from the baseline invasive coronary angiography (ICA) scan and until an endpoint occurred. All events were adjudicated by two operators (G.B, B.C) who reviewed electronic databases and documents of hospitalization or medical procedure. The study complied with the Declaration of Helsinki and was approved by all institutional ethics committees. All patients provided written informed consent or participation in the trial. The primary endpoint was a composite of death and hospilalization for heart failure.

### 2.5 Statistical analysis

Categorical data are expressed as absolute frequencies and percentages, and were compared using the χ2 test or Fisher’s exact test, as appropriate. Continuous variables are expressed as mean +/- SD for normally distributed variables and as median (25th–75th percentile) for non-normally distributed variables. Analysis was performed with the Mann–Whitney *U* test or Kruskal–Wallis test for variables with a non-normal distribution and with Student’s *t* test or analysis of variance for variables with a normal distribution. Death or readmission for heart failure in patients with and without AngioIMR > 40 was evaluated using the Kaplan-Meier method and compared among groups using the log-rank test. The index date was the date of ICA for STEMI. A multivariate Cox regression model was used to evaluate the prognostic utility of AngioIMR (as a dichotomous variable) for the primary endpoint. All individual variables listed in Table 2 with probability value of < 0.1 were considered for inclusion into multivariable forward stepwise models to determine independent predictors. In cases of collinearity, the variable with stronger prognostic importance was chosen for the multivariable analysis. To avoid multicollinearity, variables with a variance inflation factor exceeding 10 were excluded from the multivariate analysis. The discriminant function of the predictive model was evaluated using Harrell’s C statistics with 95 % CIs. Further-more, to assess the reproducibility of the measures using Angio-IMR, 20 randomly selected patients with Angio IMR analysis were evaluated twice by the same observer for intra-observer variability, and by two different observers for interobserver variability. Variability was quantified computing the intraclass correlation coefficient (ICC). Interobserver reliability for measurement of the angio-IMR was assessed by using two-way random single-measure ICC analysis. Intraobserverand intrasubject reliability was assessed by using one-way random two-measures ICC analysis. Inter and inter observer concordance for the assessment of AngioIMR > 40 was evaluated using the k test. All probability values were 2-sided, and P values < 0.05 were considered to indicate statistical significance. All statistical analysis were performed using MedCalc software version 14.8.1 (MedCalc software Ltd, Ostend, Belgium) and SPSS version 24.0.

**Table 1:** Patients’ baseline characteristics.

	**Total Population n = 178**	**AngioIMR ≤ 40** **n = 106 (60 %)**	**AngioIMR > 40** **n = 72 (40 %)**	**p value**
**Clinical characteristic**				
Age (years)	65.0 ± 12.8	63.1 ± 12.5	67.9 ± 12.8	0.01
Male sex	133 (74)	81 (76)	52 (72)	0.52
Body mass index (kg/m2)	26.2 ± 5.92	25.3 ± 5.03	27.5 ± 6.93	0.02
Hypertension n (%)	87 (48)	46 (43)	41 (57)	0.76
Diabetes mellitus	33 (18)	18 (17)	15 (21)	0.51
Dyslipidemia	59 (33)	34 (32)	25 (35)	0.71
Smoking	91 (51)	48 (45)	43 (60)	0.06
Previous MI	19 (10)	13 (12)	6 (8)	0.42
Systolic BP (mmHg)	119 ± 24	116 ± 24	124 ± 24	0.03
Heart rate (BPM)	80 ± 16	79 ± 14	82 ± 19	0.23
Total ischemic time				
<3h	91 (51)	57 (54)	34 (47)	0.39
3 to 12 h	60 (33)	36 (34)	24 (33)	0.93
>12 h	27 (15)	13 (12)	14 (19)	0.19
**Imaging measure**				
Trans thoracic echocardiography				
LVEF > 50 % at index procedure	86 (48)	61 (57)	25 (34)	0.003
Invasive coronary angiography				
Multivessel disease	63 (35)	38 (36)	25 (35)	0.87
Culprit vessel				
− LMCA	3 (2)	2 (2)	1 (1)	0.80
− LDA	90 (50)	53 (50)	37 (51)	0.85
− Left Cx	26 (14)	14 (13)	12 (17)	0.52
− RCA	59 (33)	37 (35)	22 (30)	0.54
TIMI 0 pre PCI	115 (64)	61 (57)	54 (75)	0.01
TIMI 1 pre PCI	11 (6)	8 (7)	3 (4)	0.52
TIMI 2 pre PCI	14 (8)	9 (8)	5 (7)	0.70
TIMI 3 pre PCI	38 (21)	28 (26)	10 (14)	0.04
AngioIMR	36.0 (26.9–50.0)	28.6 (23.6–33.4)	54 (47.2–65.1)	<0.0001
**Interventional treatment**				
Thrombus aspiration	55 (30)	31 (29)	24 (33)	0.56
GP Inhibitor (%)	71 (40)	35 (33)	36 (50)	0.02
Number stent deployed ≥ 2	18 (10)	8 (7)	10 (14)	0.16
TIMI 2 post PCI	8 (4)	2 (2)	6 (8)	0.06
TIMI 3 Post PCI	168 (94)	104 (98)	64 (89)	0.01

Values are mean ± SD or n (%).

MI = myocardial infarction, BP = blood pressure, BPM = beat per minute, LVEF = left ventricular ejection fraction, LMCA = left main coronary artery, LADA = left anterior descending artery, Cx = circonflex, RCA = right coronary artery, TIMI = thrombolysis in myocardial infarction, PCI = percutaneous coronary intervention, GP = glycoprotein

**Table 2:** Univariate predictors of MACE.

	**No MACE** **n = 122 (69 %)**	**MACE** **n = 56 (31 %)**	**HR** **(95 % CI)**	**p value**
**Clinical characteristic**				
Age (years)	61.4 ± 11.0	73.0 ± 13.0	1.062 (1.040–1.085)	<0.0001
Male sex	100 (82)	33 (59)	2.53 (1.490–4.325)	0.001
Body mass index (kg/m2)	26.0 ± 3.99	26.6 ± 9.12	1.012 (0.96–1.065)	0.66
Hypertension n (%)	50 (41)	37 (66)	2.038 (1.172–3.545)	0.01
Diabetes mellitus	18 (15)	15 (27)	1.181 (1.005–3.291)	0.04
Dyslipidemia	41 (34)	18 (32)	0.902 (0.515–1.582)	0.71
Smoking	56 (46)	35 (62)	1.832 (1.066–3.148)	0.02
Previous MI	11 (9)	8 (14)	1.553 (0.734–3.288)	0.25
Systolic BP (mmHg)	119 ± 24	116 ± 24	1.001 (0.989–1.013)	0.85
Heart rate (bpm)	79 ± 14	82 ± 20	1.009 (0.990–1.028)	0.35
Total ischemic time				
<3h	65 (53)	26 (46)	0.925 (0.546–1.566)	0.77
3 to 12 h	42 (34)	18 (32)	0.776 (0.442–1.362)	0.37
>12 h	15 (12)	12 (21)	1.709 (0.902–3.239)	0.1
**Imaging measure**				
Trans thoracic echocardiography				
LVEF > 50 % at index procedure	65 (53)	21 (37)	0.435 (0.251–0.754)	0.003
Invasive coronary angiography				
Multivessel disease	41 (34)	22 (39)	1.446 (0.843–2.479)	0.18
Culprit vessel				
− LMCA	1 (1)	2 (4)	4.831 (1.150–20.283)	0.03
− LDA	62 (50)	29 (52)	0.887 (0.525–1.498)	0.65
− Left Cx	17 (14)	9 (16)	1.068 (0.523–2.179)	0.85
− RCA	43 (35)	16 (29)	0.735 (0.412–1.313)	0.29
Timi0 pre PCI	78 (64)	37 (66)	1.108 (0.637–1.928)	0.71
Timi1 pre PCI	7 (6)	4 (7)	1.115 (0.403–3.083)	0.83
Timi2 pre PCI	10 (8)	4 (7)	0.874 (0.316–2.418)	0.79
Timi3 Pre PCI	27 (22)	11 (20)	0.881 (0.455–1.703)	0.70
AngioIMR ≥ 40	33 (27)	39 (70)	4.519 (2.550–8.009)	<0001
**Interventional treatment**				
Thrombo-aspiration	42 (34)	13 (23)	1.027 (0.409–2.576)	0.95
GP IIb/IIIa inhibitor (%)	50 (41)	21 (37)	0.879 (0.512–1.510)	0.64
Number stent deployed ≥ 2	12 (10)	6 (11)	1.282 (0.548–2.996)	0.56
Timi2 post PCI	5 (4)	3 (5)	1.206 (0.377–3.862)	0.75
Timi3 Post PCI	117 (96)	51 (91)	0.497 (0.198–1.245)	0.13

Values are mean ± SD or n (%).

MACE = major adverse cardiovascular event, HR = hazard ratio, MI = myocardial infarction, BP = blood pressure, BPM = beat per minute, LVEF = left ventricular ejection fraction, LMCA = left main coronary artery, LADA = left anterior descending artery, Cx = circonflex, RCA = right coronary artery, TIMI = thrombolysis in myocardial infarction, PCI = percutaneous coronary intervention, GP = glycoprotein

## 3 Results

### 3.1 Study population

The clinical, imaging and interventional procedure data are reported in Table 1. The mean age was 65 years with 74 % of male gender. The total ischemic time was less than 3 h in almost 50 % of cases. The left ventricular ejection fraction (LVEF) on the initial transthoracic echocardiography was preserved in one out of two cases. Infarction was anterior in half of the cases. Restoration of TIMI 3 flow was achieved in 168 patients (94 %). The mean AngioIMR was 36 U (interquartile 26.9–50). Patients with AngioIMR > 40U were significantly older, with a rarer initially preserved LVEF. Initial coronary occlusion, GP IIbIIIa inhibitors use and failure to restore TIMI 3 flow were also more frequent. During a median follow-up of 2.9 (2.3–6.9) years, the primary endpoint was observed in 56 patients (31 %). All-cause death was observed in 52 patients (29 %) while hospitalization for heart failure occurred in 4 patients (2 %).

### 3.2 Prognostic value

**Fig. 2:**
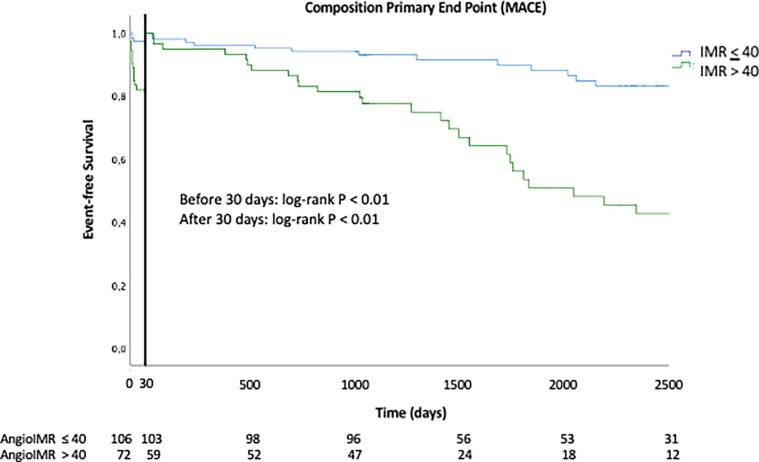
**Landmark analysis according angioIMR** Kaplan–Meier curves with the threshold of 30 days (landmark analysis) for progression-free survival. The primary end point was all-cause death or hospitalization for heart failure; MACE = Major adverse cardiovascular events; IMR = Index of microcirculatory resistance.

The variables associated with MACE in univariate and multivariate analyses are represented in Table 2, Table 3, respectively. An AngioIMR > 40 was significantly associated with the occurrence of the primary endpoint (70 % vs 27 %; hazard ratio 4.519; 95 % CI: 2.550–8.009; p < 0.0001) independently of other variables (Cox analysis, hazard ratio 4.282; 95 % CI: 2.325–7.886; p < 0.0001). In addition to the elevation of AngioIMR, the multivariate analysis also revealed poor prognostic factors, including older age, female gender, and the presence of the culprit lesion in the left main coronary artery. Kaplan-Meier analysis demonstrated varying trends in survival without death or hospitalization for heart failure over time based on the presence or absence of an elevated AngioIMR. The two curves exhibited significant divergence (p < 0.001), with an initial substantial gap followed by a progressively widening separation in the second phase (Fig. 2).

**Table 3:** Independent Predictors for Cardiac Death or Readmission for Heart Failure by multivariate Cox Analysis.

	**HR (95 % CI)**	**p value**
Age	1.049 (1.023–1.075)	0.0001
Male sex	2.082 (1.156–3.750)	0.01
Hypertension	1.387 (0.764–2.519)	0.28
Diabetes	1.950 (1.026–3.705)	0.04
Smoking	1.583 (0.848–2.956)	0.14
LVEF > 50 % at index procedure	0.742 (0.394–1.397)	0.35
LMCA	14.87 (3.068–72.13)	0.001
AngioIMR > 40	4.517 (2.434–8.381)	0.0001

HR = hazard ratio, LVEF = left ventricular ejection fraction, LMCA = left main coronary artery.

### 3.3 Incremental prognostic value

**Fig. 3:**
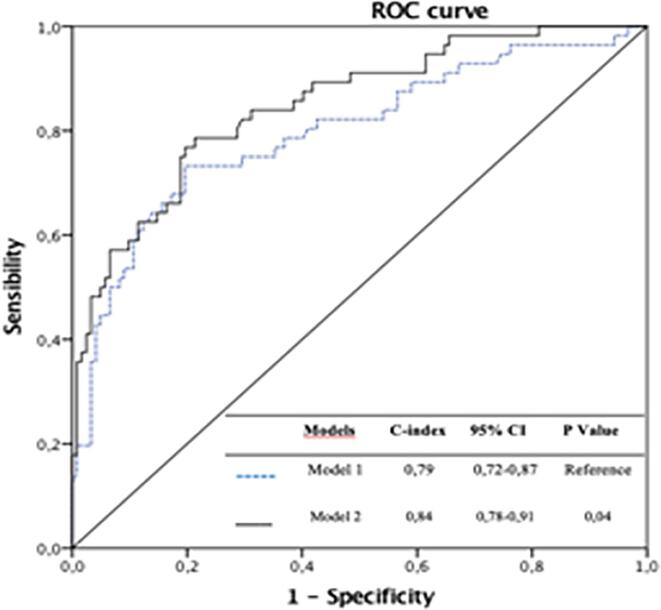
**ROC Curve with incremental prognostic value of AngioIMR compared to other multivariate predictors of MACE***Model 1 = clinical and imaging risk predictors (multivariate predictors of MACE) Model 2 = Model 1 + AngioIMR* ROC = Receiver operating characteristic; MACE = Major adverse cardiovascular events.

The integration of AngioIMR into a logistic regression model combining all variables present in the multivariate analysis indicated its incremental prognostic value as demonstrated by the significant increase in diagnostic performance (C-index 0.84 vs 0.79 respectively with and without integration of angio-IMR; p = 0.04) (Fig. 3).

### 3.4 Reproducibility of angioIMR

Intra and inter observer reproducibility of angio-IMR was good as depicted in Table 4. intra and interobserver concordance for AngioIMR > 40 in patients were k = 0.89 and 0.37, respectively.

**Table 4:** Reproducibility of angioIMR.

	Intra-observer reproducibility		Inter-observer reproducibility	
	Coefficient of variation %	ICC	Coefficient of variation %	ICC
AngioIMR	30.4	0.92 (0.80–0.96)	34.8	0.86 (0.66–0.94)

## 4 Discussion

The present study evaluated the prognostic value of AngioIMR in patients with PPCI for STEMI. The main result was that AngioIMR was an independent prognostic marker of all cause mortality or hospitalization for heart failure.

### 4.1 IMR and angiography derived IMR: Theranostic biomarker?

CMD in STEMI as assessed by invasive IMR is a powerful prognostic factor [13]. Consequently, it has been adopted as a theranostic biomarker of CMD in various studies aimed at advancing stratified medicine for STEMI. A theragnostic biomarker is a metric that predicts therapeutic response. Compared with other invasive and non invasive indexes, IMR has more validation data, suggesting a stronger potential for implementation into clinical practice [20]. However, the deployment of a thermodilution wire during STEMI presents numerous drawbacks, notably that it is not the primary choice of wire in this clinical scenario, primarily due to issues related to trackability and hydrophobic coating. The possibility of angiography-derived IMR is very attractive. Several indexes have been described, each with variations in their measurement methods (Table 5) [21], [22], [23]. The first measurement of an angiography-derived IMR was performed by De Maria et al. by ​​modifying the IMR formula with the introduction of quantitative flow reserve (QFR) instead of fractional flow reserve (FFR). While this measurement has demonstrated its prognostic significance, it necessitates the induction of hyperemia [24]. After discontinuing the use of the pressure wire, the subsequent simplification of the procedure involved eliminating the need for hyperemia, which required the modeling of hyperhemic Pa from rest Pa. This was achieved, for instance, through the caIMR formula, where the estimation of hyperemic Pa was calculated as Mean Aortic Pressure (MAP) multiplied by 0.2 or 0.15, depending on whether MAP is less than 95 mmHg or greater than 95 mmHg. The prognostic value of this angiography derived IMR was studied by Choi et al. in 309 patients with STEMI. The results indicated an excellent correlation between invasive IMR and CaIMR in the diagnostic cohort, and a significant prognostic value for AngioIMR > 40 in the prognostic cohort. Indeed, sensitivity, specificity, accuracy, and area under the curve of caIMR for the prediction of an IMR > 40 U were respectively 75.0 %, 84.2 %, 80.6 %, and 0.899 (95 % confidence interval: 0.786–0.949), and the hazard ratio for all cause death and hospitalization for heart failure were 2.173 (95 % CI: 1.157–4.079; P = 0.016) [25]. Another possibility used by NH-angioIMR is to take the resting pressure without assuming a hyperemia-induced pressure variation. In a population of patients with acute or chronic coronary syndromes, a better correlation was observed between NH-angioIMR and invasive IMR in STEMI patients [26]. This suggested that the drop in Pa was lower in the context of STEMI, which could be explained by an alteration of coronary vasodilator properties due to thrombosis and ischemia–reperfusion injury [27]. Nevertheless, a ∼ 10 % reduction in aortic pressure is reported in hyperemia, both in coronary artery disease at large [18] but also in STEMI [28].

**Table 5:** Review of formulas used to derive IMR from angiography.

**Authors / Study**	**Clinical presentation**	**Hyperemia**	**Formulas**		**Vendors**
De Maria et al., 2020	STEMI	Yes	Pa x QFR x $\frac{\mathrm{Nframes}}{\mathrm{Fps}}$	IMRangio	Medis
Scarsini et al., 2021	STEMI/ NSTEMI/ CCS	No		NH-IMRangio	
Kotronias et al., 2021	STEMI	No		NH-IMRangio	
Fan et al,. 2022	ACS and CCS	No	$\frac{Pax\mu QFR}{\mathrm{Vhyp}}$	AMR	Pulse Medical
Tebaldi et al., 2020	CCS	No	(Pa x $\mathrm{vessel}\frac{\mathrm{length}}{\mathrm{Flowvelocity}}$ x (1,35 x cQFR) – 0,32)/100	A-IMR	Medis
Choi et al., 2021	STEMI	No	Pa x caFFR x $\mathrm{vessel}\frac{\mathrm{length}}{\mathrm{KxVdiastole}}$	caIMR	RainMed
Mejia-Renteria et al., 2021	CCS	No	(Pa(rest) – (0,1 x Pa (rest))) x QFR x $\mathrm{vessel}\frac{\mathrm{length}}{\mathrm{Vhyp}}$	AngioIMR	Medis
Fan et al.,2023Zhang et al., 2024	STEMI, NSTEMI and CCSNSTEMI	YesYes	Pahyp x accuFFRhyp x L/ Vhyp	AccuIMR	ArteryFlow

ACS = acute coronary syndrome; A-IMR = angio-derived index of microcirculatory resistance; AMR = angiographic microvascular resistances; caIMR = coronary angiography-derived index of microvascular resistance; CCS = chronic coronary syndrome; Fps = frame per second; k = kappa constant; NH = non hyperhemic; NSTEMI = non ST elevation myocardial infarction; Pa = aortic pressure; Pd = distal pressure, QFR = quantitative flow reserve; STEMI = ST elevation myocardial infarction; V = velocity, Vhyp = velocity modeled in hyperemia.

### 4.2 First demonstration of AngioIMR prognosis value

Our study is the first to evaluate the prognostic value of AngioIMR by Medis Medical Imaging Systems as determined using angiographic images and resting aortic pressure. As indicated in the introduction section, the formula assumes a hyperemic drop in Pa of ∼10 %. Our findings indicated that the use of AngioIMR allows improved patient stratification, particularly for those with an adverse prognosis, even in cases where epicardial revascularization was deemed satisfactory. While our study was the first to assess the prognostic value of angioIMR of Medis medical imaging by MACE analysis, a recent study has demonstrated its diagnostic value, with a correlation with invasive IMR in a STEMI population (*r* = 0.70, *p <* 0.001), and a good predictive value of MVO (AUC 0.79 (95 % CI: 0.667–0.928)) [29]. It is important to demonstrate the diagnostic and prognostic value of these new indices, as CMD presented prognosis value in other cardiovascular conditions beyond STEMI, such as chronic coronary syndromes, non-ST elevation myocardial infarction, heart failure preserved ejection fraction, and cardiomyopathies [30]. The 4 companies that provide angiography derived IMR use the same basic principles with use 3D reconstruction of a vessel from angiographic projections, the contrast flow velocity derived from the length of the vessel divided by the contrast filling time, and then converted into hyperemic flow velocity. Finally, pressure drop is calculated, based on fluid dynamic equations with the above-mentioned hyperemic flow as the boundary condition. Raimed technology uses a real-time aortic pressure measurements taken by a specific device (FlashPressure, Rainmed Ltd.) to measure caFFR. The recorded aortic pressure is averaged over five cardiac cycles and converted to hyperemic aortic pressure using a simple formula. AMR by Pulse Medical Technology uses μQFR with a novel computational approach based on Murray’s law. 3D quantitative coronary angiography is reconstructed with only a single angiographic projection. In this computation, an average aorta pressure of 86 mmHg during maximum hyperemia is assumed. The latest technology, AccuIMR by ArteryFlow technology, uses a classic methodology with no special features compared to the Medis system, with the exception of 3D coronary modeling [31], [32]. However, while CaIMR, AMR and AccuIMR respectively use caFFR, μQFR and accuFFR in their equation, these indices are less validated compared to QFR, which currently has the highest level of validation as shown by the latest recommendations [33].

Similar to conventional IMR, AngioIMR could serve as a valuable instrument in advancing stratified medicine, targeting costly or invasive interventions specifically for selected patients. The detection and treatment of CMD in the spectrum of cardiovascular diseases is the subject of growing interest and is part of a stratified, even personalized medicine [30]. This is particularly true in STEMI [20], [34], [35]. For example, in anterior STEMI, supersatured oxygen (SSO2) therapy has demonstrated its benefits for the reduction of infarct size and improvement in cardiac remodeling by action on CMD [36]. Nevertheless, SSO2 therapy presents certain limitations, necessitating the establishment of a new femoral vascular access in cases where the procedure is conducted through radial access, and mandates the catheterization laboratory to be immobilized for a duration of 60 min (not including the associated costs of devices and consumables). Currently in our structure we are leading the IC-HOT-Micro study, the objective of which is to research the effects of SSO2 therapy on AngioIMR (NCT05790876), with the aim of developing a stratified medicine strategy guided by AngioIMR.

## 5 Limitations

Our study has limitations. It is a single-centered study with a relatively small sample size, and there was no measurement of invasive IMR to establish a robust correlation. Second, the primary limitation of this study is the presence of selection bias. The AIDECORO cohort comprises STEMI patients with high-quality angiographic images used for training a deep learning artificial intelligence algorithm. This cohort as an event rate higher than expected probably due to this selection biais. Consequently, the findings may not be universally generalizable, and prospective interventional clinical trial which used AngioIMR to a stratified medicine will be necessary.

## 6 Conclusion

In this study of patients with STEMI, an AngioIMR > 40 predicted a significantly higher risk for all-cause death or hospitalization for heart failure. In addition to well-known risk factors, assessment of coronary microvascular dysfunction can be a feasible approach for early prevention and a therapeutic target in STEMI patients.

## CRediT authorship contribution statement

**Benoit Caullery:** Writing – review & editing, Writing – original draft, Software, Formal analysis. **Laurent Riou:** Writing – review & editing. **Stephanie Marliere:** Writing – review & editing, Visualization. **Estelle Vautrin:** Writing – review & editing, Visualization. **Nicolas Piliero:** Writing – review & editing, Visualization. **Olivier Ormerzzano:** Writing – review & editing, Visualization. **Helene Bouvaist:** Writing – review & editing, Visualization. **Gerald Vanzetto:** Writing – review & editing, Visualization. **Gilles Barone-Rochette:** Writing – review & editing, Writing – original draft, Methodology, Investigation, Formal analysis, Data curation, Conceptualization.

## Declaration of competing interest

The authors declare that they have no known competing financial interests or personal relationships that could have appeared to influence the work reported in this paper.
